# Multi-dimensional data transmission using inverse-designed silicon photonics and microcombs

**DOI:** 10.1038/s41467-022-35446-4

**Published:** 2022-12-21

**Authors:** Ki Youl Yang, Chinmay Shirpurkar, Alexander D. White, Jizhao Zang, Lin Chang, Farshid Ashtiani, Melissa A. Guidry, Daniil M. Lukin, Srinivas V. Pericherla, Joshua Yang, Hyounghan Kwon, Jesse Lu, Geun Ho Ahn, Kasper Van Gasse, Yan Jin, Su-Peng Yu, Travis C. Briles, Jordan R. Stone, David R. Carlson, Hao Song, Kaiheng Zou, Huibin Zhou, Kai Pang, Han Hao, Lawrence Trask, Mingxiao Li, Andy Netherton, Lior Rechtman, Jeffery S. Stone, Jinhee L. Skarda, Logan Su, Dries Vercruysse, Jean-Philippe W. MacLean, Shahriar Aghaeimeibodi, Ming-Jun Li, David A. B. Miller, Dan M. Marom, Alan E. Willner, John E. Bowers, Scott B. Papp, Peter J. Delfyett, Firooz Aflatouni, Jelena Vučković

**Affiliations:** 1 E.L.Ginzton Laboratory, Stanford University, Stanford, CA USA; 2 John A. Paulson School of Engineering and Applied Sciences, Harvard University, Cambridge, MA USA; 3 The College of Optics and Photonics, University of Central Florida, Orlando, FL USA; 4 Time and Frequency Division, National Institute of Standards and Technology, Boulder, CO USA; 5 Department of Physics, University of Colorado, Boulder, CO USA; 6 Department of Electrical and Computer Engineering, University of California, Santa Barbara, CA USA; 7 Department of Electrical and Systems Engineering, University of Pennsylvania, Philadelphia, PA USA; 8 SPINS Photonics Inc, Hollister, CA USA; 9 Octave Photonics, Louisville, CO USA; 10 Department of Electrical and Computer Engineering, University of Southern California, Los Angeles, CA USA; 11 Applied Physics Department, The Hebrew University of Jerusalem, Jerusalem, Israel; 12 Corning Incorporated, Corning, NY USA

**Keywords:** Integrated optics, Frequency combs, Nanophotonics and plasmonics

## Abstract

The use of optical interconnects has burgeoned as a promising technology that can address the limits of data transfer for future high-performance silicon chips. Recent pushes to enhance optical communication have focused on developing wavelength-division multiplexing technology, and new dimensions of data transfer will be paramount to fulfill the ever-growing need for speed. Here we demonstrate an integrated multi-dimensional communication scheme that combines wavelength- and mode- multiplexing on a silicon photonic circuit. Using foundry-compatible photonic inverse design and spectrally flattened microcombs, we demonstrate a 1.12-Tb/s natively error-free data transmission throughout a silicon nanophotonic waveguide. Furthermore, we implement inverse-designed surface-normal couplers to enable multimode optical transmission between separate silicon chips throughout a multimode-matched fibre. All the inverse-designed devices comply with the process design rules for standard silicon photonic foundries. Our approach is inherently scalable to a multiplicative enhancement over the state of the art silicon photonic transmitters.

## Abstract (web summary)

The authors demonstrate a multi-dimensional communication scheme that combines wavelength- and mode- multiplexing on photonic integrated circuits using foundry-compatible photonic inverse design and spectrally flattened microcombs

## Introduction

Chip-scale optical interconnects^1,2^ are primarily developed with the wavelength-division multiplexing (WDM) technique, which enables parallel signal transmission by independently encoding data on multiple frequencies of light^3–6^. To further increase the link bandwidth, other promising dimension of signal encoding that can be utilised for multiplexing is the spatial domain; light can be decomposed into a set of optical beams with orthogonal spatial cross-sections. These orthogonal spatial modes in multimode optical waveguides or in free space can serve as independent communication channels^7–15^, each of which can support a full WDM link. This orthogonality gives mode-division multiplexing (MDM) a multiplicative effect on the bandwidth of an optical link. Recently, significant progress has been made towards integrating mode and wavelength-division multiplexing together on a chip^16–25^.

In this work, we present a multi-wavelength, multimode communication scheme for on-chip and chip-to-chip interconnects. Using photonic inverse design^26,27^, we implement a low-crosstalk, all-passive silicon MDM that supports parallel WDM channels over a 15-THz spectral bandwidth. With this device, we demonstrate a 1.12-Tb/s error-free data transmission on a silicon photonic circuit using microcombs^28,29^ as multi-wavelength laser sources: 28 data channels derived from seven wavelength channels launched into four spatial mode channels. The MDM device also enables spectrally efficient WDM due to its non-resonant operation. Additionally, in combination with inverse-designed beam emitters and a multimode-matched fibre^30,31^, we demonstrate chip-to-chip data transmission across four spatial mode channels between separate silicon chips. In contrast to the previous work^22,23^, the photonic inverse design of the MDM components is fully compatible with commercial foundries for the fabrication of photonic-electronic systems, and exhibits low loss in large bandwidth necessary to demonstrate full communication systems.

## Results

### A silicon multi-dimensional transmitter

**Fig. 1:**
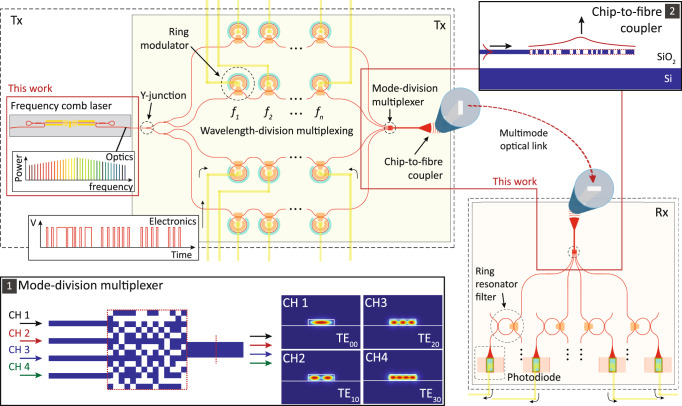
An optical transmitter using the multi-dimensional multiplexing principle of chip-to-chip WDM-MDM data transmission. A frequency comb laser is evenly distributed to individual WDM transmitters (e.g. cascaded ring modulators^39^) using two-stage Y-junction splitters. The multi-frequency data from each WDM transmitter are routed into spatial modes of a multimode waveguide using an MDM multiplexer (Inset 1). The optical data can then be transmitted through chip-to-fibre couplers and multimode fibre to the receiver, where the mode and wavelength channels are separated by MDM-WDM demultiplexers and detected using photodiodes. The chip-to-fibre coupler is optimised to emit multimode beams in the surface-normal direction and improve coupling efficiencies with orthogonal spatial modes of multimode fibre (Inset 2).

The schematic of the multi-wavelength, multimode optical interconnect is illustrated in Fig. 1. A multi-wavelength laser source^4,28,32–38^ is evenly distributed into multiple WDM transmitter circuits, and each WDM circuit independently encodes data onto different frequencies of light^39,40^. An inverse-designed MDM multiplexer takes the overlapping modes from multiple WDM transmitters and transforms them into co-propagating spatially orthogonal modes of a multimode optical waveguide. The MDM multiplexer routes the fundamental transverse-electric mode (TE_00_) of the input single-mode waveguides to the TE_00_ (channel 1), TE_10_ (channel 2), TE_20_ (channel 3) and TE_30_ (channel 4) modes of an output multimode waveguide (Inset 1 of Fig. 1). A chip-to-chip optical link enables the transmission of the WDM-MDM data between separate silicon chips throughout free space or optical fibre. A receiver circuit de-multiplexes the spatially and spectrally mapped signals and converts the data to electronic circuits via photodetectors.

**Fig. 2:**
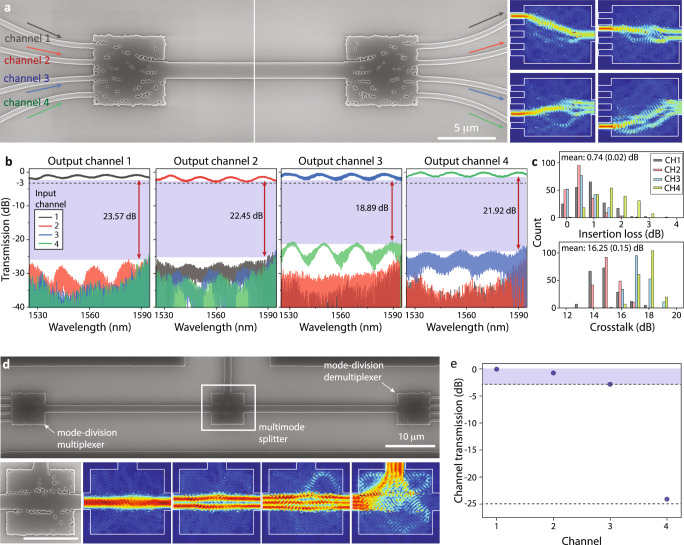
Inverse design of silicon multimode photonics. **a–c** Broadband, low-crosstalk MDM multiplexers: **a** SEM images of back-to-back MDM multiplexer devices with simulated mode conversion (right). **b** Measured transmission of the back-to-back MDM multiplexer structure. This broadband structure is utilized in the WDM-MDM data transmission experiments (see Fig. 3). **c** Insertion loss and crosstalk histograms of the back-to-back MDM multiplexers fabricated in a semiconductor foundry^41^. **d**, **e** Multimode splitter which combines a waveguide bend (for TE_30_) and waveguide crossing (for TE_00_, TE_10_ and TE_20_): **d** Top: SEM image of a multimode photonic circuit which consists of MDM multiplexer, multimode splitter, and MDM demultiplexer (from left to right). The multimode splitter (white rectangular box) selectively routes multimode signals to different directions. Bottom: Zoomed-in SEM image and simulated spatial mode routing. **e** Measured channel transmission for the multiplexer-splitter-demultiplexer structure (wavelength: 1540 nm).

The inverse-designed MDM devices were fabricated in a standard silicon-on-insulator platform (silicon thickness: 220 nm). Figure 2a shows a scanning electron micrograph (SEM) image of the MDM muliplexer, whose design area is 6.5 × 6.5 μm^2^ with a foundry-compatible minimum feature size (80 nm)^41^. The input and output single-mode waveguides are 500-nm-wide, and the multimode waveguide between multiplexers is 1800-nm-wide. The device structure is optimised for operations between wavelengths of 1500 and 1600 nm, and Fig. 2b shows measured channel transmissions of the back-to-back MDM multiplexers. We find that the peak insertion loss is less than 0.8- and 3-dB bandwidth is wider than 120 nm for all mode channels (Fig. S1c). The modal crosstalk, which must be suppressed to avoid MDM signal degradation, is less than −18 dB for all mode channels. The crosstalk of the broadband design is significantly lower compared to that of the narrow band design (see Fig. S1). Broadband optimisation has previously been shown to be an effective heuristic for robustness to fabrication errors^42,43^. In addition, Fig. 2c presents insertion loss and crosstalk histograms of channel transmission measurements from six different MDM designs. For this analysis, the inverse-designed structures were fabricated in GlobalFoundries^41^ and the measured histograms are from the back-to-back MDM multiplexers at multiple wavelengths between 1540 and 1560 nm.

Our MDM multiplexers exhibit low insertion loss in a large bandwidth and a compact footprint, all necessary to demonstrate full communication systems. It is enabled by photonic inverse design that can combine multiple directional couplers and adiabatic waveguide tapers in a single compact device—typically, multiple sets of these two device components build a single MDM multiplexer and take up a significant device footprint. The design approach is scalable to larger spatial mode channels (e.g. 6 and 12 mode channels in Figs. S2 and S3, respectively), and we find empirically that the device footprint needed for high-performance MDM structures is approximately proportional to the number of spatial mode channels. Furthermore, we can combine other device functionalities in a compact footprint using optimisations. As an example, Fig. 2d, e present an inverse-designed multimode splitter that provides selective device operations for different spatial mode channels—90^∘^ waveguide bend for TE_30_, and waveguide crossing for TE_00_, TE_10_ and TE_20_. We connect MDM multiplexers along with the multimode splitter in series, and characterise the device operation: measured channel transmission losses of the multiplexer-splitter-demultiplexer circuit are lower than 3 dB, and transmission suppression as a result of waveguide bending (CH4: TE_30_) is up to 24 dB.

### On-chip interconnect

In this section, we show an on-chip optical interconnect using the silicon photonic MDM and microcombs. The microcomb spectrum is engineered to improve a communication link capacity^28,29^, and we compare data transmission results of anomalous- and normal-dispersion Kerr soliton microcombs. The experiments allow us to rigorously understand an operational limit of the foundry-compatible MDM with chip-scale lasers for a full photonic-electronic system implementation.

**Fig. 3:**
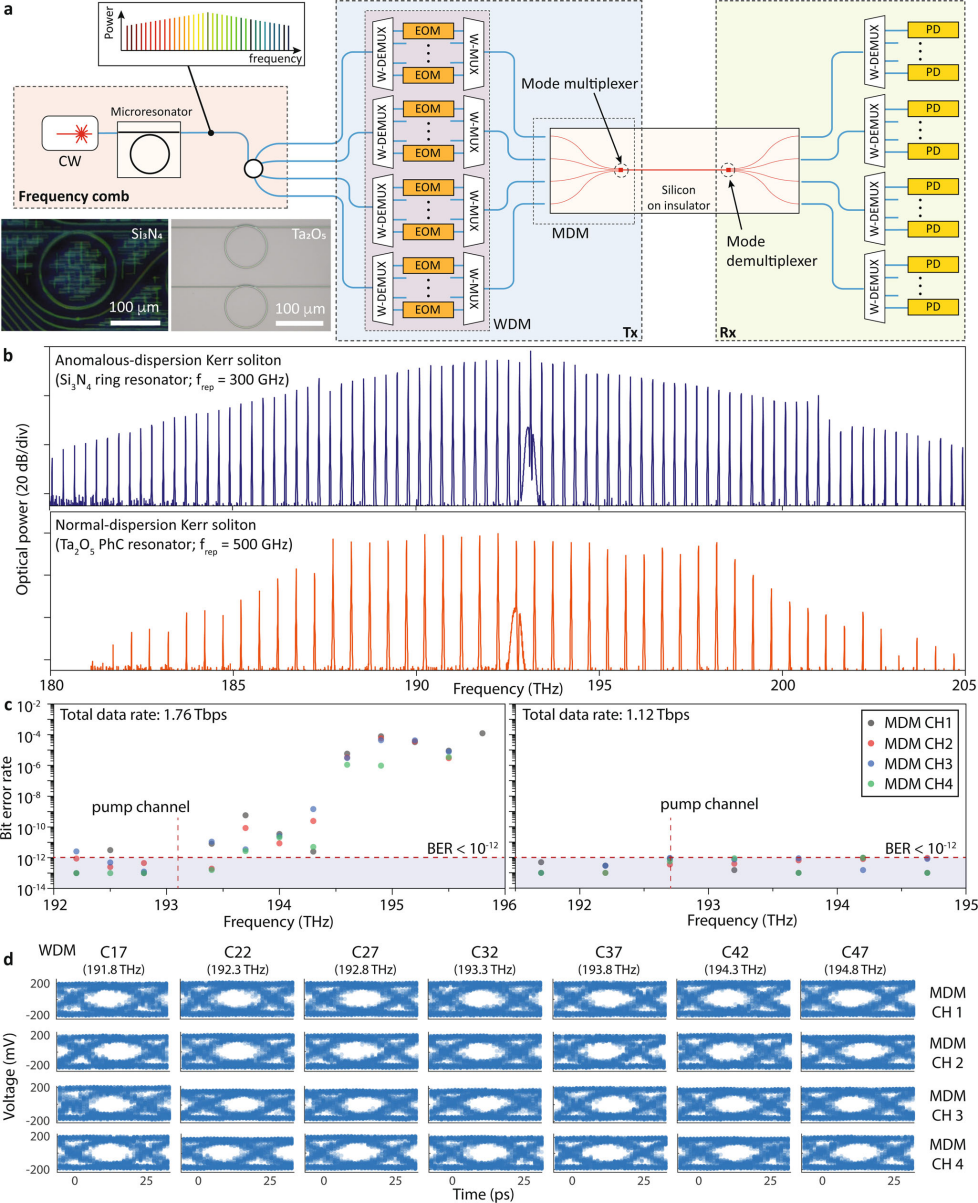
On-chip interconnect: WDM-MDM data transmission with microcombs. **a** Data transmission scheme using inverse-desiged MDM and microcombs. The transmitter optical source is generated by pumping the microresonator (Inset: optical microscope images of Si_3_N_4_ microresonator and Ta_2_O_5_ photonic crystal ring resonator^28,29,37^) with a CW laser. WDM de-multiplxers (W-DEMUX) separate the comb lines and intensity modulators (EOM) encode independent data (NRZ at a symbol rate of 40 GBd/channel). The WDM data were recombined using WDM multiplexers (W-MUX), and are coupled to on-chip MDM inputs simultaneously using a fibre array. At the receiver, all the channels are separated by mode and wavelength demultiplexers and detected using a photodiode (PD). In our experiments, we independently modulate even and odd WDM channels using two EOMs for emulating WDM transmission (see Method section). **b** Optical spectra of anomalous- and normal-dispersion Kerr soliton combs with Si_3_N_4_ and Ta_2_O_5_ resonators, respectively. For the data transmission measurements, 11 and 7 comb lines at the C band were used. **c** Measured BERs (10^12^ bits compared) of the transmitted data channels. For those with BER <10^−12^, we counted an error occurrence with up to 10^13^ reference bits. **d** 40-Gb/s eye diagrams of Ta_2_O_5_ combs data channels directly detected using PD.

The conceptual diagram of the on-chip data transmission is presented in Fig. 3a. Microcombs are generated as circulating pulses in an optical cavity pumped with a CW laser, leading to low-noise, broadband, multi-wavelength light sources^4,28,29,37,40^. We launch 11 and 7 WDM channels into four MDM channels using microcombs with 300-GHz and 500-GHz frequency spacings (Fig. 3b), respectively. The 300-GHz-comb is generated in an anomalous-dispersion Si_3_N_4_ ring resonator, and the spectral envelope of the comb exhibits a secant-squared profile. The 500-GHz-comb is generated in a Ta_2_O_5_ photonic crystal ring resonator that enables comb generations under normal dispersion (see method for more detail), resulting in higher conversion efficiency, optical power per comb line, and spectral flatness^28,29,40^. All data channels are directly detected using external photodiodes, and individual channels are encoded with non-return-to-zero (NRZ) data coding at a symbol rate of 40 GBd. We use external electro-optic modulators and a 100-GHz-grid WDM in this experiment. The external components increase the total system transmission loss, and optical amplifiers (see Fig. S6) are used to compensate for the extra losses. The extra losses can be mitigated once all components are integrated, and the MDM components are compatible with foundry processes, and can be fully integrated with chip-scale WDM transmitters^3,39,40^.

Bit-error rates (BERs) of the data transmission are presented in Fig. 3c. First, of the 52 carriers derived from four spatial modes and the Si_3_N_4_ combs^37^ in the C band, 44 data channels were used for data transmission, resulting in a total line rate of 1.76 Tb/s. 11 channels show no error occurrences when a total of 10^12^ bits are compared (BER <10^−12^), and another 15 channels show error rates between 10^−12^ and 10^−8^. Data channels with higher carrier frequencies show larger error rates than other wavelength channels, owing to lower comb line power and higher amplifier noise at those WDM channels. The pump tone at ~193.1 THz couldn’t be used for data transmission as well. For the transmission experiment using a spectrally flattened Ta_2_O_5_ combs^28,29^, 28 data channels were derived from four spatial modes and all the comb lines in the C band. In contrast to the Si_3_N_4_ combs, all of them were used for data transmission without an error occurs when a total of 10^12^ bits are compared, as a result of the engineered comb spectrum. The total net data rate is 1.12 Tb/s, and Fig. 3d presents eye diagrams of all data channels.

In a single MDM device, which can achieve a spectral bandwidth of over 70 nm (Fig. 2), further improvement in the data transmission capacity is straightforward through the use of adjacent S and L bands. We can also decrease the frequency spacing of the comb source to the standard 100-GHz frequency grid^4^ or even finer^36^. As a proof of concept, we demonstrate WDM-MDM data transmission (4-mode, 16-wavelength/mode) using a mode-locked laser with a 20-GHz channel spacing. Fig. S7 shows multi-dimensional data transmission with spectral efficiency of 4 bit/s/Hz and BER of all channels lower than the threshold of 20% hard-decision error correction. A total line rate is 1.28 Tb/s only with 0.32 THz of the WDM spectral bandwidth.

### Chip-to-chip multimode optical link

**Fig. 4:**
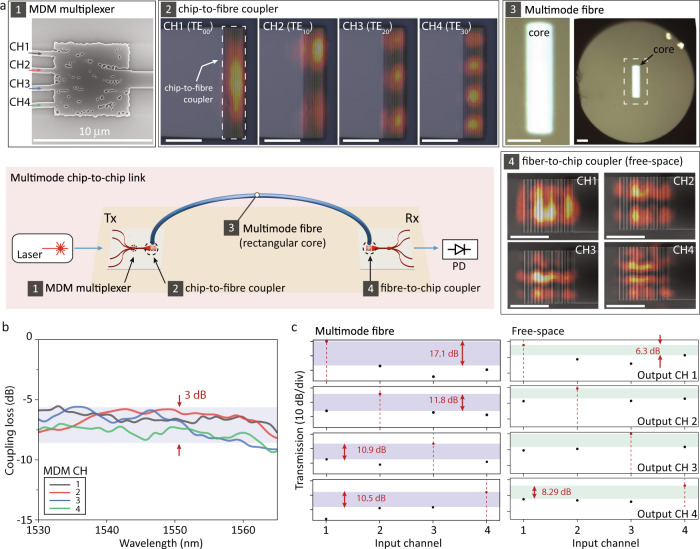
Chip-to-chip multimode link using inverse-designed couplers. **a** Schematic of a multimode link (scale bars: 10 μm). A CW laser is coupled to an input single-mode waveguide of the Tx chip via a lensed fibre. The input signal is routed into a specific spatial mode of a multimode waveguide using an MDM multiplexer (Inset 1). Inverse-designed chip-to-fibre coupler emits the multimode signal in the surface-normal direction (Inset 2: Infrared images of multimode beams at the top surface of the device are overlaid on device SEM images), and the multimode beams can be coupled to spatial modes of a multimode-matched fibre (Inset 3: Microscope image of the fibre cross-section). The MDM signal then is sent to the Rx chip, and routed into a specific single-mode output waveguide of the chip throughout fibre-to-chip coupler (Inset 4: multimode beams on the coupler. Infrared images are overlaid on device SEM images, and the multimode beams are from the Tx chip throughout free-space optic alignment) and MDM demultiplexer. All data channels are directly detected using photodiodes at the receiver side. **b** Measured coupling loss spectra of the fibre-to-chip coupler for spatial mode channels. For this measurement, transmitted power was measured through MDM multiplexer, chip-to-fibre coupler, rectangular core fibre and photodetector. To estimate coupling losses, the insertion loss of the MDM multiplexer and transmission loss of the fibre were subtracted. **c** Measured chip-to-chip MDM channel crosstalks at 1540 nm (Left: multimode fibre chip-to-chip link, Right: free-space).

In this section, we demonstrate a chip-to-chip multimode link using inverse-designed couplers and a rectangular core multimode fibre^31^, shown schematically in Fig. 4a. The chip-to-chip interconnect requires efficient means of coupling light from on-chip transceivers to a communication link. In both free-space and fibre systems, this can be achieved using a grating coupler^20,44–46^ that preserves and launches all spatial modes perpendicular to the chip.

Using the photonic inverse design, we can optimise structures to form any desired spatial field patterns^47^ with multiple design objectives (e.g. operational bandwidth^48^, incident angle, and back reflection). In this experiment, the structure is optimised to improve coupling efficiencies with orthogonal spatial modes of multimode fibres at multiple wavelengths over 40 nm spectral span. Simultaneously, the structure is designed to emit multimode beams in a surface-normal direction with a minimum feature size of 80 nm. Furthermore, it is designed to suppress back-reflections from the silicon chip to spatial modes of the multimode fibre when the signal is coming from the fibre to the chip. The back-reflections are usually related to the thickness of the buried oxide layer, and the inverse-designed structure can further reduce the backward direction coupling to spatial modes of the fibre.

A CW laser is coupled to the Tx chip through one of four single-mode input waveguides via a lensed fibre. The Tx chip then sends light into a specific spatial mode of the multimode fibre using the MDM multiplexer (Inset 1 of Fig. 4a) and inverse-designed coupler (Inset 2 of Fig. 4a). The 5-metre-long multimode fibre, which has a rectangular core (dimension: 32 × 8 μm^2^; Inset 3 of Fig. 4a) constrained to support a single mode in the narrow transverse direction and four modes in the orthogonal direction, transmits the signal from the Tx chip to the Rx chip while maintaining low modal crosstalk. The Rx chip de-multiplexes the multimode signals using fibre-to-chip coupler (Inset 4 of Fig. 4a) and MDM demultiplexer, and we individually characterise the transmitted signals at single-mode output waveguides.

Figure 4b shows the measured coupling loss of the inverse-designed coupler. A 3-dB-bandwidth of the chip-to-fibre coupling is over 35 nm for all mode channels, and this is at least two times larger compared to prior results^24^. We can potentially design structures for wider bandwidth operations^48^. The mode-dependent loss difference is less than 2.5 dB for all spatial mode channels over 35 nm spectral bandwidth, and the coupling loss of the MDM channel 4 (TE_30_) is slightly higher than other channels, mainly due to fibre-to-chip alignment. It is important to note that the coupling loss of the multimode coupler isn’t lower than those of the standard couplers with fully etched silicon layer^48^ and the coupling efficiency can be significantly improved with partially etched silicon layer or foundry-compatible bi-level gratings.

Figure 4c shows the measured channel crosstalks of the chip-to-chip MDM with the rectangular core fibre. For comparison, we also conduct a free-space experiment where multimode signals are transmitted between chips using free-space optics (see the experimental setup and data transmission results in Figs. S8, S9) and the link crosstalk is only attributed to the free-space beam alignment. MDM channel 4, the most difficult channel to align, shows the highest crosstalks with all other spatial mode channels in the free-space link measurement. The multimode fibre link shows lower crosstalks (−10.5 to −17.1 dB) compared to the free-space link as a result of an improved alignment, but it still shows higher crosstalks compared to the back-to-back MDM multiplexer measurements due to modal crosstalks in the multimode fibre. The communication link capacity, in terms of total transmission loss and crosstalks, can be further improved with advanced photonic packaging for both MDM links with multimode fibre and free-space optics^49,50^.

## Discussion

In summary, we have demonstrated a multi-dimesional optical communication scheme using silicon photonic circuits. With an inverse-designed silicon MDM device and chip-scale microcombs, we multiplex four spatial modes and comb channels covering the entire C band to achieve natively error-free (<10^−12^) data transmission through a nanophotonic waveguide. Additionally, we show chip-to-chip multimode links enabled by inverse-designed couplers and a rectangular core multimode fibre. The MDM technology is fully compatible with silicon photonic WDM transmitters and semiconductor foundry processes. Photonic inverse design ensures device compactness and broadband operation. This enhances robustness to fabrication errors and temperature fluctuations, enabling practical applications for high-performance silicon chips.

Scaling of this technology for ultra-wide bandwidth high-fidelity communication will require the maintenance of low-mode crosstalk and insertion loss. In particular, we observe modal crosstalks in chip-to-chip data transmission experiments. This signal degradation can be compensated by using MZI meshes^51^ or digital MIMO signal processing^21,52,53^. Future scaled-up implementations of our communication scheme can employ such error-correction optical circuits on the receiver chip, and their performance can be improved in terms of footprint, insertion loss and operation bandwidth using photonic inverse design. Furthermore, efficient edge couplers^54,55^, foundry-compatible bi-level gratings^56,57^, as well as advanced fibre technologies^58^ can further mitigate mode crosstalk and insertion loss.

As our MDM device features non-resonant, low-loss, low-crosstalk operation over the entire C band, it is compatible with a large number of novel light sources, not limited to microcombs (Fig. 4) and a table-top mode-locked laser (Fig. S7) demonstrated in this work. For example, different types of chip-scale lasers and frequency comb sources such as mode-locked quantum dot lasers^5^, electro-optical frequency combs^34,59^, and vertical cavity surface emitting laser (VCSEL)^60^ can be used. Co-design of the laser source, optical link architecture, photonic devices and signal processing will facilitate the next generation of optical interconnects in data-centre networks and hardware accelerators.

## Methods

### Photonic inverse design

Stanford Photonics Inverse Design Software (SPINS)^61,62^ was used to design an MDM multiplexer, mode-splitter and surface-normal grating couplers for TE-polarised light.

### Si_3_N_4_ microcombs

Anomalous-dispersion microcomb is generated by pumping a Si_3_N_4_ microring resonator with a CW laser, IQ modulator in a single-sideband suppressed-carrier configuration driven by a voltage-controlled oscillator for a fast wavelength tuning, and a subsequent amplifier^37^. Figure S6 presents the comb generation setup with a silicon nitride device. The output of the comb is passed through a WDM filter to suppress CW pump power.

### Ta_2_O_5_ microcombs

Normal-dispersion microcomb is generated by pumping a Ta_2_O_5_ photonic crystal ring resonator with a CW pump laser and fibre amplifier—this comb state doesn’t require a fast laser frequency tuning using an external modulator. The inner wall of the photonic crystal ring resonator is modified by an azimuthally uniform, periodic modulation pattern to open a bandgap only at a targeted azimuthal mode (approximately at 1555 nm) that we excite with the pump laser^28,29^. Both forward and backward propagating comb can exist in the photonic crystal ring resonator, and we use a backward propagating comb with higher pump-to-comb conversion efficiency in this experiment. We collect the comb signal using a fibre circulator and suppress CW pump power using a tunable fibre filter.

### WDM-MDM data transmission

Comb signals are de-multiplexed using a commercial 100-GHz ITU grid-based DWDM with an insertion loss of 3.5 dB per channel. The ‘even’ and ‘odd’ carriers are recombined using another multiplexer. Each channel is separately amplified and passed through two intensity modulators (6-dB insertion loss) which are driven by PRBS31 generators using NRZ at a data rate of 40 Gb/s. The data channels are de-correlated with a delay of ~20,000 symbols. We recombine the odd and even sets of carriers, amplify them using an EDFA and split the power into four different channels. Mode channel 2–4 have a delay of 200, 400 and 800 symbols with respect to mode channel 1. Output coupling is performed with a lensed fibre aligned to one MDM output waveguide at a time. The light at the output is then amplified and sent through another demultiplexer, where the signal is detected using a commercial photodiode. The photodetected signal is then sent to an error analyzer which we use to measure the bit-error rate. Figure S6 shows a detailed experimental setup.

### Four spatial mode rectangular core fibre

An optical fibre with a rectangular core geometry was provided by Corning Incorporated. The germanium-doped core material was machined to a rectangular shape and with an annular silica cladding material was drawn down to final dimensions (125 μm cladding diameter, nearly perfectly circular, with a core of 32 × 8 μm^2^ and index modulation ~0.005). The fibre supports four polarisation-degenerate spatial modes with intermode effective index differences ≥0.0005. A 5-metre-long fibre sample was used to image the four guided modes using low-coherence interferogram image analysis (beating against a plane wave, see Fig. S10). The measured differential modal group delay was ~8 ns/km, which can lead to signal skew in long mode multiplexed links and MIMO processing complexity if mode mixing occurs.

## Supplementary information

Supplementary Information

## Data Availability

All the data generated in this study are available from the corresponding author upon reasonable request.

## Code availability

An open-source of the photonic optimisation software is available at https://github.com/stanfordnqp/spins-b.

## References

[1] Miller DA (2009). Device requirements for optical interconnects to silicon chips. Proc. IEEE.

[2] Miller DA (2017). Attojoule optoelectronics for low-energy information processing and communications. J. Lightwave Technol..

[3] Sun C (2015). Single-chip microprocessor that communicates directly using light. Nature.

[4] Marin-Palomo P (2017). Microresonator-based solitons for massively parallel coherent optical communications. Nature.

[5] Liu S (2019). High-channel-count 20 GHz passively mode-locked quantum dot laser directly grown on Si with 4.1 Tbit/s transmission capacity. Optica.

[6] Raja, A. S. et al. Ultrafast optical circuit switching for data centers using integrated soliton microcombs. *Nat Commun* **12**, 5867 (2021). 10.1038/s41467-021-25841-8 PMC8520010 34654810

[7] Miller DA (2019). Waves, modes, communications, and optics: a tutorial. Adv. Opt. Photonics.

[8] Wang J (2012). Terabit free-space data transmission employing orbital angular momentum multiplexing. Nat. Photonics.

[9] Richardson D, Fini JM, Nelson LE (2013). Space-division multiplexing in optical fibres. Nat. Photonics.

[10] Bozinovic N (2013). Terabit-scale orbital angular momentum mode division multiplexing in fibers. Science.

[11] Ryf R (2011). Mode-division multiplexing over 96 km of few-mode fiber using coherent 6 × 6 MIMO processing. J. Lightwave Technol..

[12] Van Uden RG (2014). Ultra-high-density spatial division multiplexing with a few-mode multicore fibre. Nat. Photonics.

[13] Kahn JM, Miller DA (2017). Communications expands its space. Nat. Photonics.

[14] Puttnam, B. et al. 0.61 Pb/s S, C, and L-band transmission in a 125*μ*m diameter 4-core fiber using a single wide-band comb source. *J. Lightwave Technol*. **39**, 1027–1032 (2020).

[15] Rademacher G (2021). Peta-bit-per-second optical communications system using a standard cladding diameter 15-mode fiber. Nat. Commun..

[16] Gabrielli LH, Liu D, Johnson SG, Lipson M (2012). On-chip transformation optics for multimode waveguide bends. Nat. Commun..

[17] Luo L-W (2014). WDM-compatible mode-division multiplexing on a silicon chip. Nat. Commun..

[18] Miller SA (2020). Large-scale optical phased array using a low-power multi-pass silicon photonic platform. Optica.

[19] Dai D (2018). 10-channel mode (de) multiplexer with dual polarizations. Laser Photonics Rev..

[20] Baumann, J. M. et al. Silicon chip-to-chip mode-division multiplexing. In *2018 Optical Fiber Communications Conference and Exposition* (*OFC*) 1–3 (IEEE, 2018).

[21] Huang, H. et al. Demonstration of terabit coherent on-chip optical interconnects employing mode-division multiplexing. *Opt. Lett.* **46**, 2292–2295 (2021). 10.1364/OL.424727 33988567

[22] Frellsen LF, Ding Y, Sigmund O, Frandsen LH (2016). Topology optimized mode multiplexing in silicon-on-insulator photonic wire waveguides. Opt. Express.

[23] Chang W (2018). Ultra-compact mode (de) multiplexer based on subwavelength asymmetric Y-junction. Opt. Express.

[24] Tong Y, Zhou W, Wu X, Tsang HK (2019). Efficient mode multiplexer for few-mode fibers using integrated silicon-on-insulator waveguide grating coupler. IEEE J. Quantum Electron..

[25] Hu H (2018). Single-source chip-based frequency comb enabling extreme parallel data transmission. Nat. Photonics.

[26] Piggott AY (2015). Inverse design and demonstration of a compact and broadband on-chip wavelength demultiplexer. Nat. Photonics.

[27] Molesky S (2018). Inverse design in nanophotonics. Nat. Photonics.

[28] Yu S-P (2021). Spontaneous pulse formation in edgeless photonic crystal resonators. Nat. Photonics.

[29] Zang, J., Yu, S.-P., Carlson, D. R., Briles, T. C. & Papp, S. B. Near unit efficiency in microresonator combs. In *CLEO: Science and Innovations*, STh4F-3 (Optica Publishing Group, 2022).

[30] Blau M, Marom DM (2019). Wavelength demultiplexer designs operating over multiple spatial modes of a rectangular waveguide. IEEE J. Sel. Top. Quantum Electron..

[31] Rechtman, L., Marom, D. M., Stone, J. S., Peng, G. & Li, M.-J. Mode characterization of rectangular core fiber. In *2017 IEEE Photonics Conference* (*IPC*) 43–44 (IEEE, 2017).

[32] Koenig S (2013). Wireless sub-THz communication system with high data rate. Nat. Photonics.

[33] Hu H (2018). Single-source chip-based frequency comb enabling extreme parallel data transmission. Nat. Photonics.

[34] Zhang M (2019). Broadband electro-optic frequency comb generation in a lithium niobate microring resonator. Nature.

[35] Corcoran B (2020). Ultra-dense optical data transmission over standard fibre with a single chip source. Nat. Commun..

[36] Shen B (2020). Integrated turnkey soliton microcombs. Nature.

[37] Briles TC, Yu S-P, Drake TE, Stone JR, Papp SB (2020). Generating octave-bandwidth soliton frequency combs with compact low-power semiconductor lasers. Phys. Rev. Appl..

[38] Jørgensen, A. et al. Petabit-per-second data transmission using a chip-scale microcomb ring resonator source. *Nat. Photonics* **16**, 1–5 (2022).

[39] Wade, M. et al. An error-free 1 Tbps WDM optical I/O chiplet and multi-wavelength multi-port laser. In *Optical Fiber Communication Conference*, F3C-6 (Optical Society of America, 2021).

[40] Rizzo, A. et al. Integrated kerr frequency comb-driven silicon photonic transmitter. Preprint at arXiv:2109.10297 (2021).

[41] Rakowski, M. et al. 45nm CMOS-silicon photonics monolithic technology (45CLO) for next-generation, low power and high speed optical interconnects. In *Optical Fiber Communication Conference*, T3H-3 (Optical Society of America, 2020).

[42] Piggott AY (2020). Inverse-designed photonics for semiconductor foundries. ACS Photonics.

[43] Yang KY (2020). Inverse-designed non-reciprocal pulse router for chip-based lidar. Nat. Photonics.

[44] Su T (2012). Demonstration of free space coherent optical communication using integrated silicon photonic orbital angular momentum devices. Opt. Express.

[45] Cai X (2012). Integrated compact optical vortex beam emitters. Science.

[46] Tong Y, Zhou W, Wu X, Tsang HK (2019). Efficient mode multiplexer for few-mode fibers using integrated silicon-on-insulator waveguide grating coupler. IEEE J. Quantum Electron..

[47] White, A. D. et al. Inverse Design of Optical Vortex Beam Emitters. *ACS Photonics*10.1021/acsphotonics.2c01007 (2022).

[48] Sapra, N. V. et al. Inverse design and demonstration of broadband grating couplers. In *IEEE Journal of Selected Topics in Quantum Electronics* (IEEE, 2019).

[49] De Heyn, P. et al. Ultra-dense 16x56 Gb/s NRZ GeSi EAM-PD arrays coupled to multicore fiber for short-reach 896 Gb/s optical links. In *Optical Fiber Communication Conference*, Th1B-7 (Optical Society of America, 2017).

[50] Dietrich P-I (2018). In situ 3D nanoprinting of free-form coupling elements for hybrid photonic integration. Nat. Photonics.

[51] Annoni A (2017). Unscrambling light-automatically undoing strong mixing between modes. Light Sci. Appl..

[52] Arik SÖ, Kahn JM (2016). Direct-detection mode-division multiplexing in modal basis using phase retrieval. Opt. Lett..

[53] Huang H (2015). Mode division multiplexing using an orbital angular momentum mode sorter and mimo-dsp over a graded-index few-mode optical fibre. Sci. Rep..

[54] Gordillo, O. A. J., Dave, U. D. & Lipson, M. Bridging between si and few-mode fiber higher order modes. In *CLEO: Science and Innovations*, SM2O-6 (Optical Society of America, 2020).

[55] Shen W, Du J, Xiong J, Ma L, He Z (2020). Silicon-integrated dual-mode fiber-to-chip edge coupler for 2 × 100 Gbps/lambda MDM optical interconnection. Opt. Express.

[56] Su L (2018). Fully-automated optimization of grating couplers. Opt. Express.

[57] Notaros, J. et al. Ultra-efficient CMOS fiber-to-chip grating couplers. In *2016 Optical Fiber Communications Conference and Exhibition* (*OFC*) 1–3 (IEEE, 2016).

[58] Puttnam BJ, Rademacher G, Luís RS (2021). Space-division multiplexing for optical fiber communications. Optica.

[59] Idjadi, M. H., Arab, S. & Aflatouni, F. Optical frequency comb generation in silicon by recursive electro-optic modulation. In *CLEO: Science and Innovations*, SF3O-5 (Optical Society of America, 2020).

[60] Chen, H. et al. 10-Mode-Multiplexed Transmitter Employing 2-D VCSEL Matrix. In *2021 European Conference on Optical Communications* (*ECOC*) 1–4 (IEEE, 2021).

[61] Vuckovic, J. et al., Inverse design software for nanophotonic structures—spins. *Stanford University* https://stanford.resoluteinnovation.com/technologies/S18-012 (2018).

[62] Su L (2020). Nanophotonic inverse design with spins: Software architecture and practical considerations. Appl. Phys. Rev..

