# Supplementary material for: Multi-dimensional data transmission using inverse-designed silicon photonics and microcombs
Source: Nat Commun. 2022 Dec 21;13:7862. doi: 10.1038/s41467-022-35446-4 (PMC9772188; doi:10.1038/s41467-022-35446-4)
Supplement: Supplementary file 1 — Supplementary Information [file 41467_2022_35446_MOESM1_ESM.pdf]

Supplementary section for
Multi-dimensional data transmission using inverse-designed silicon photonics and microcombs

I. PHOTONIC INVERSE DESIGN OF MDM MULTIPLEXER

Broadband MDM designs, and robustness to fabrication error Broadband optimization has previously been shown to be an effective heuristic for a robustness to fabrication errors, and we test two different inverse-designed structures optimized for narrow- (Fig.S1a) and broad-band (Fig.S1b) operations. Fig. S1 shows measured channel transmissions of the back-to-back MDM multiplexers. The broadband design (panel b) exhibits lower insertion loss and cross-talk compare to those of the narrow band design.

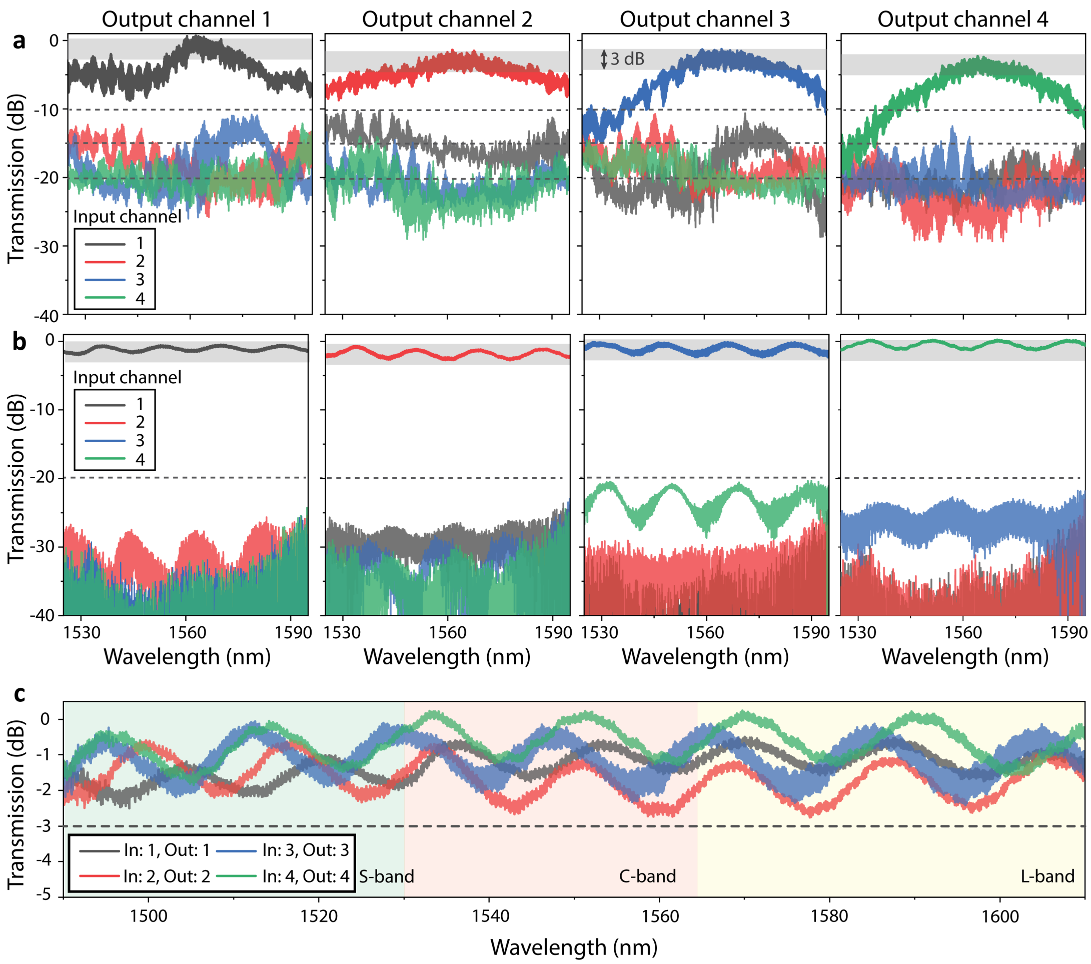

FIG. S1. **Optimization of broadband, low-crosstalk MDM multiplexer.** Measured channel transmissions of the back-to-back MDM multiplexer structures. The structure in (**b**) is optimized at multiple wavelengths over the entire C band, while the structure in (**a**) is optimized only at a single wavelength (1560 nm). The narrow band structure is implemented in the WDM-MDM communications using a mode-locked laser (16 wavelength channels over 320 GHz spectral span; see Fig.S7), and the broadband structure is utilized in the WDM-MDM communications using soliton microcombs (13 wavelength channels over 3.6 THz spectral span; see Fig.3). (**c**) Zoom-in scan of communication channel transmissions over 120 nm wavelength span.

Scalability to more spatial modes Inverse design approach is scalable to larger spatial mode channels, and the device footprint is approximately proportional to the number of spatial mode channels. Footprints of MDM multiplexers with 2 (Ref.[1]), 4 (Fig.2), 6 (Fig.S2), and 12 (Fig.S3) spatial mode channels are 3.55 × 2.55, 6.5 × 6.0, 9.0 × 9.0, and 18 × 18 μm^2, respectively. The MDM structures are optimized with full three-dimensional simulations. Only for the 12 channel MDM, we restrict our study to a two-dimensional structure for computational simplicity.

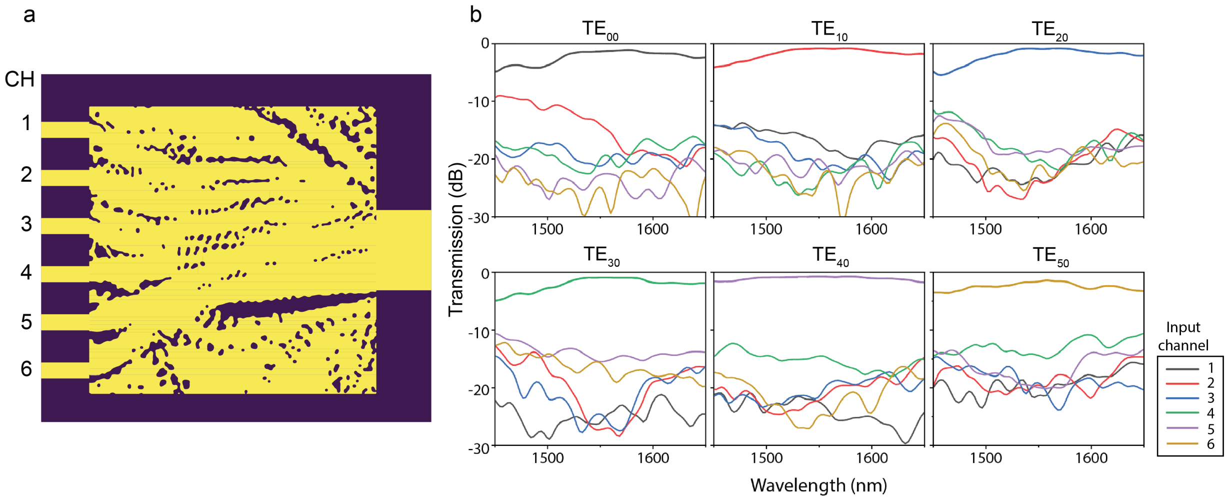

FIG. S2. **Optimization of 6 channel mode-division multiplexer.** **(a)** Structure of optimized device (9 μm × 9 μm), which consists of six single-mode input waveguides and one multimode output waveguide **(b)** Simulated channel transmission spectra.

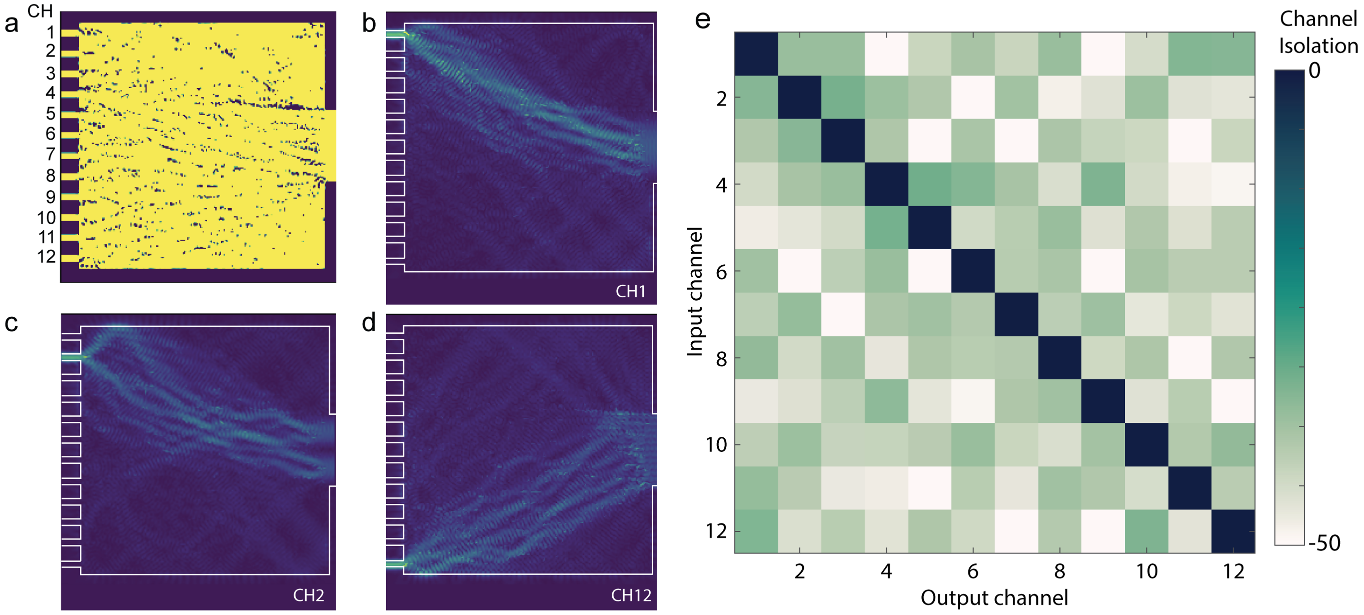

FIG. S3. **Optimization of 12 channel mode-division multiplexer.** For computational simplicity, we restrict our study to a two-dimensional structure with transverse-electric polarization. We model the structure with infinite extent in the third dimension and an effective relative permittivity of a silicon slab (220-nm-thick silicon at 1550 nm wavelength). **(a)** Structure of optimized device (18 μm × 18 μm), which consists of twelve single-mode input waveguides and one multimode output waveguide **(b-d)** Mode 1-, 2- and 12- transmission through device. **(e)** S-parameters of simulated device (note that it is a single multiplexer device and it is not a simulation of back-to-back structure). The channel crosstalk ranges from -53 dB to -25 dB, and insertion loss ranges from from -0.23 dB to -0.47 dB.

II. FOUNDRY-COMPATIBLE MDM COMPONENTS

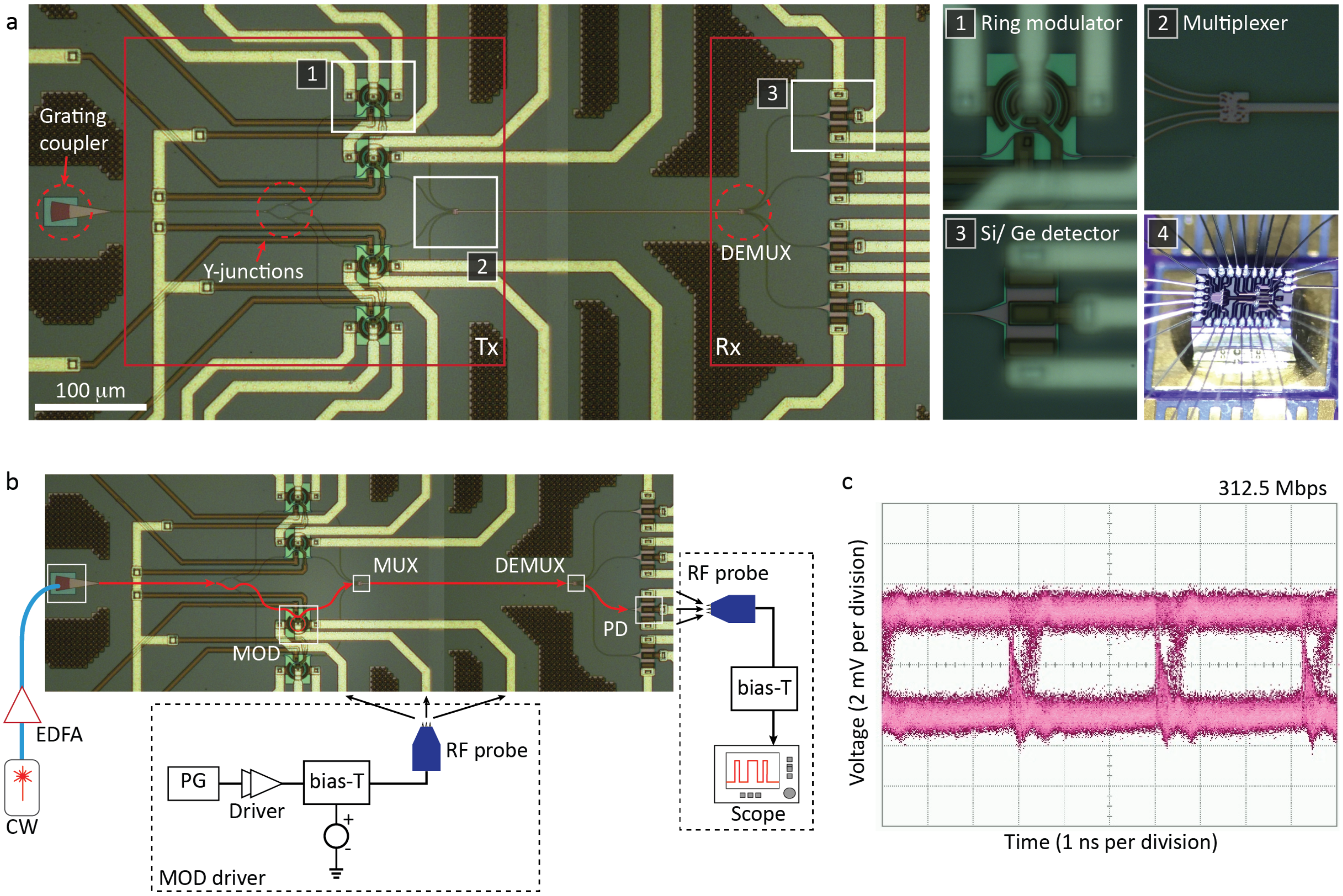

FIG. S4. **Integrated MDM transceiver.** (**a**) Microscope image of integrated four channel MDM transceiver (Inset 1: ring modulator, Inset 2: MDM multiplexer, Inset 3: Si/Ge photodetector, Inset 4: wire-bonded chip). (**b**) Data transmission measurement schematic. (**c**) Measured eye diagram of MDM channel 3 (TE_{20}) obtained from an integrated Si/Ge photodiode (PD) and external signal scope. A single wavelength signal was transmitted throughout one spatial mode channel at a time. We confirm that back reflections at the foundry-fabricated MDM and other sources of channel degradation do not limit the signal quality for further communication experiments.

The AMF photonic process is a 180 nm silicon-on-insulator fabrication process with 2 μm buried oxide thickness. The thickness of the silicon layer is 220 nm and the single-mode (500 nm width) photonic waveguides have propagation loss of about 2 dB/cm. The grating coupler is designed by partially etching silicon and has a measured loss of about 4 dB[2]. Also, the Y-junction has a measured excess loss of 0.5 dB[3]. The implemented PN ring modulator has a free spectral range of about 11 nm and a 3-dB bandwidth of higher than 30 GHz[2]. The photodiode has a responsivity of higher than 0.75 A/W and a 3-dB bandwidth of more than 30 GHz[2].

Fig.S4 shows measured results of data transmission from electro-optic ring modulator to MDM components and Si/Ge photodiode. A CW laser is coupled to an integrated MDM transceiver (GC: grating coupler, MOD: ring modulator, PG: pattern generator, MUX/DEMUX: MDM multiplexer/demultiplexer, PD: photodiode). The cascaded Y-junctions evenly distribute optical power and simultaneously send a light to four ring modulators – one of ring modulators is driven by an external electric driver. For the eye diagram measurement, one mode channel at one wavelength is transmitted with lower microwave drive frequency (312.5 Mb/s). This is because the tape-out chip has only two metal layers and it is challenging to avoid high-frequency electronic cross-talks even with an optimized layout. In addition, we characterize an optical channel isolation of the back-to-back MDM multiplexer on the AMF chip and the measured cross-talks range from -14.7 dB to -25.5 dB.

To further verify a foundry-compatibility, Fig.S5 shows measured channel transmissions of back-to-back MDM multiplexers fabricated in AIM Photonics and Global Foundries. We sent inverse-designed structures with single- and multi-wavelength optimizations to AIM photonics and Global Foundries, respectively.

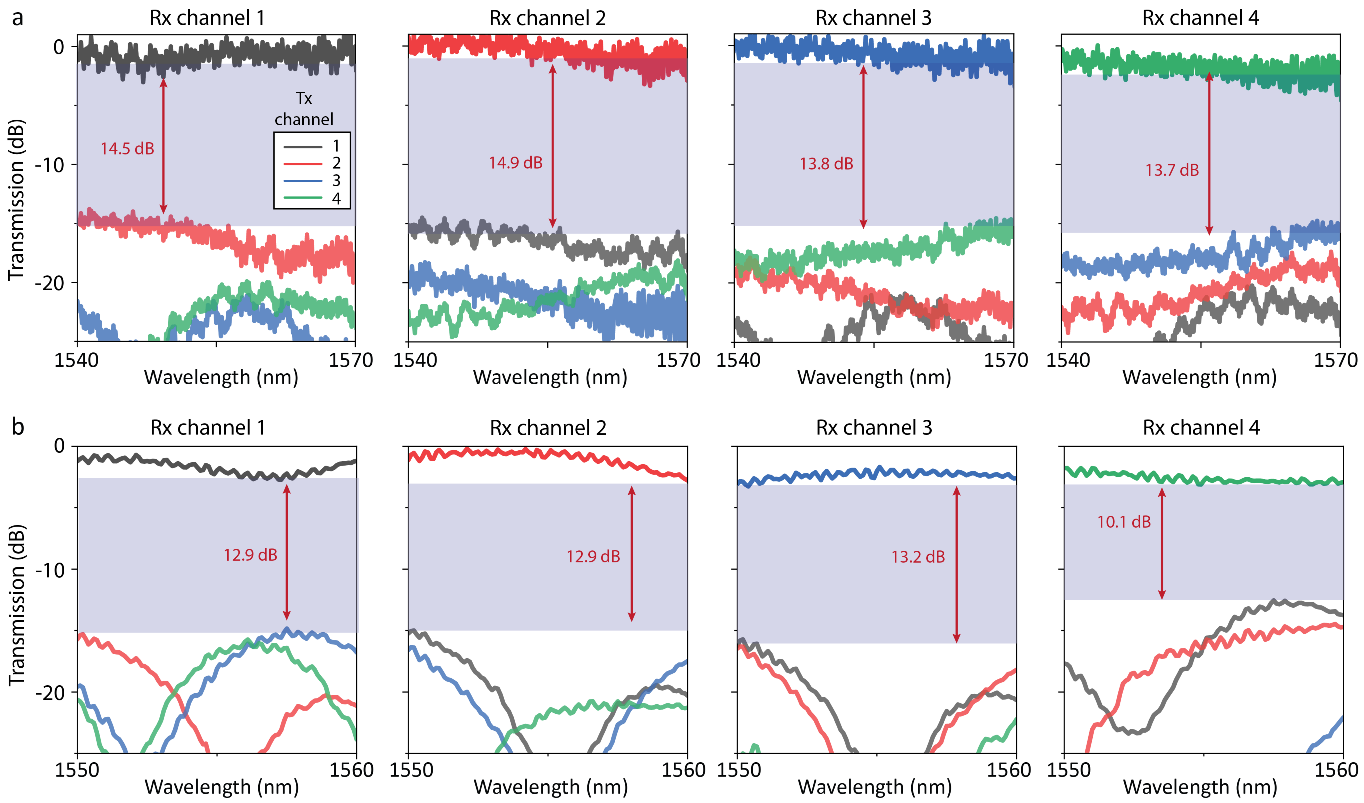

FIG. S5. **Characterization of back-to-back MDM multiplexers.** Measured channel transmissions of the back-to-back MDM multiplexer structures fabricated in (**a**) GlobalFoundries and (**b**) AIM Photonics. Optimization wavelengths: 1500, 1540, 1580, 1620 nm for GlobalFoundreis; 1555 nm for AIM Photonics.

III. DATA TRANSMISSION EXPERIMENTS WITH FREQUENCY COMBS

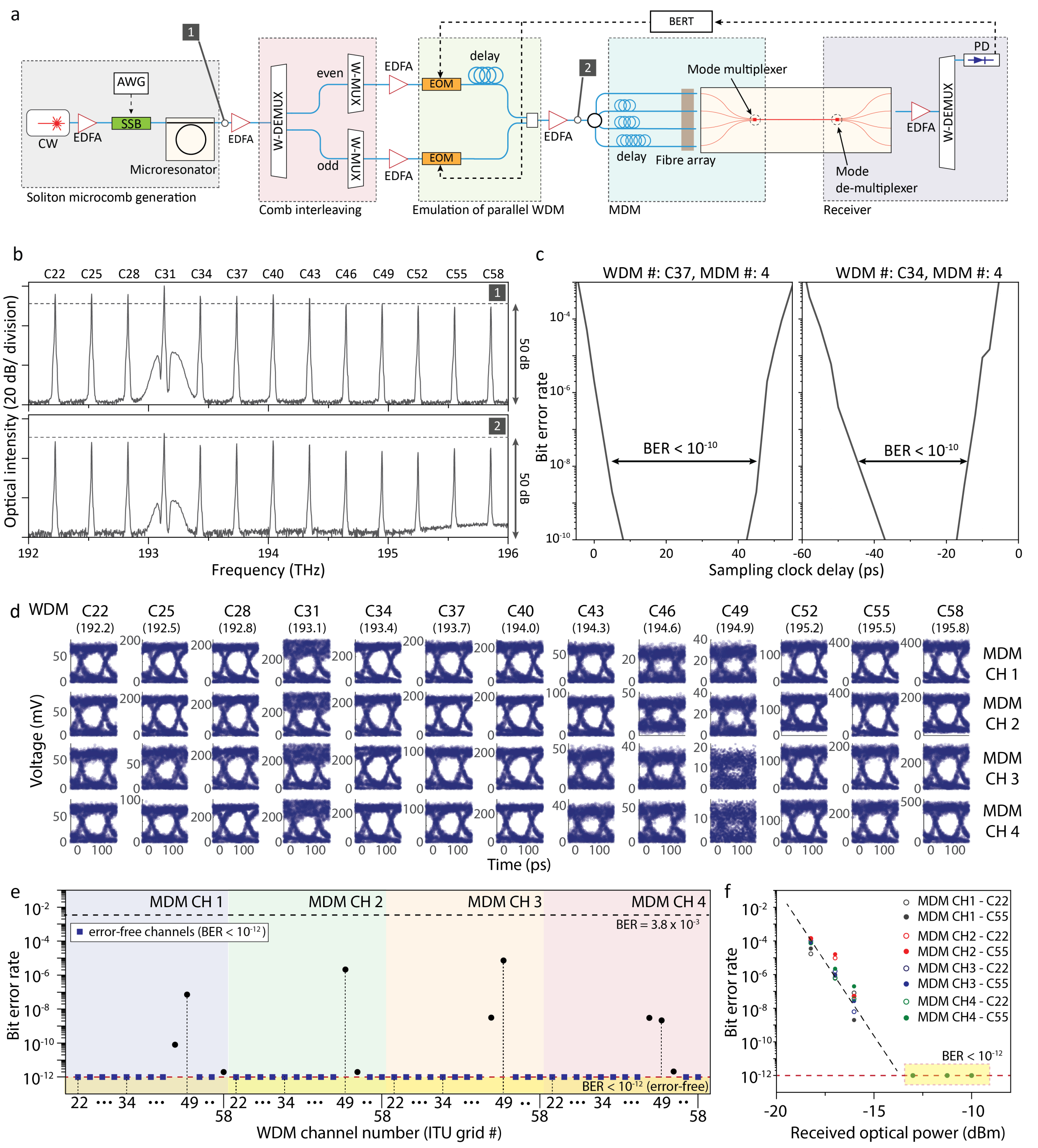

FIG. S6. **Microcomb data transmission.** **(a)** Schematic of experimental setup. **(b)** Frequency comb spectra before (top panel) and after (bottom panel) optical amplification. **(c)** 10-Gb/s bathtub curves obtained from BERT by sweeping the delay between the clocks for transmitter and receiver. Left: Measured curves of the channel C37 (193.7 THz)/ M4 (TE_{30}), Right: Bathtub curves of the channel C34 (193.4 THz)/ M4 (TE_{30}). **(d)** 10-Gb/s eye diagrams of all data channels directly detected using PD. **(e)** Measured BERs (10^{12} bits compared) of the transmitted data channels, and **(f)** BER sensitivity versus received optical power.

Microcomb source In Fig.S6, a silicon nitride microresonator generates a frequency comb with a 300 GHz repetition rate (optical spectrum: top panel of **(b)**). The comb lines are de-multiplexed using a standard ITU grid DWDM (W-DEMUX), and de-interleaved into ‘odd’ and ‘even’ carriers using another multiplexer (W-MUX). The ‘odd’ and ‘even’

channels are amplified and passed through intensity modulators (EOM) which are driven by PRBS generator using a NRZ encoding. The data channels are decorrelated by a fibre delay and re-combined into a standard single-mode fibre (optical spectrum: bottom panel of **(b)**). The combined sets of carriers are amplified and simultaneously coupled to four single-mode waveguide inputs of the silicon MDM chip (see Fig.S1b), and the transmitted light through the MDM chip is coupled back to single-mode fibre using a lensed fibre. The collected light is amplified and sent through another demultiplexer (W-DEMUX) where the received signal is analyzed using a photodiode (PD) and an error analyzer.

For this section, we present data transmission experiments with a rate of 10 Gb/s per data channel using the 300-GHz Si_3N_4 microcombs. The eye diagrams and bit error rates (BERs) of the microcomb data transmission experiment are presented in Fig.S6c-f. Out of the 52 carriers derived from the four spatial mode channels and all comb lines in the C band, 42 data channels show no error occurrences when a total of 10^{12} bits are compared (BER $< 10^{-12}$). The data channels also show BERs better than 10^{-10} with -13 dBm input optical power at 10 Gb/s/channel (see Fig.S6c and Fig.S6f). The total data rate in this experiment amounts to 520 Gb/s. In this experiment, data channels at specific WDM channels (e.g. C46 and C49) show larger error rates than other wavelength channels, owing to lower comb line power at the WDM channel and non-uniformity of the soliton optical spectrum (see Fig.S6b).

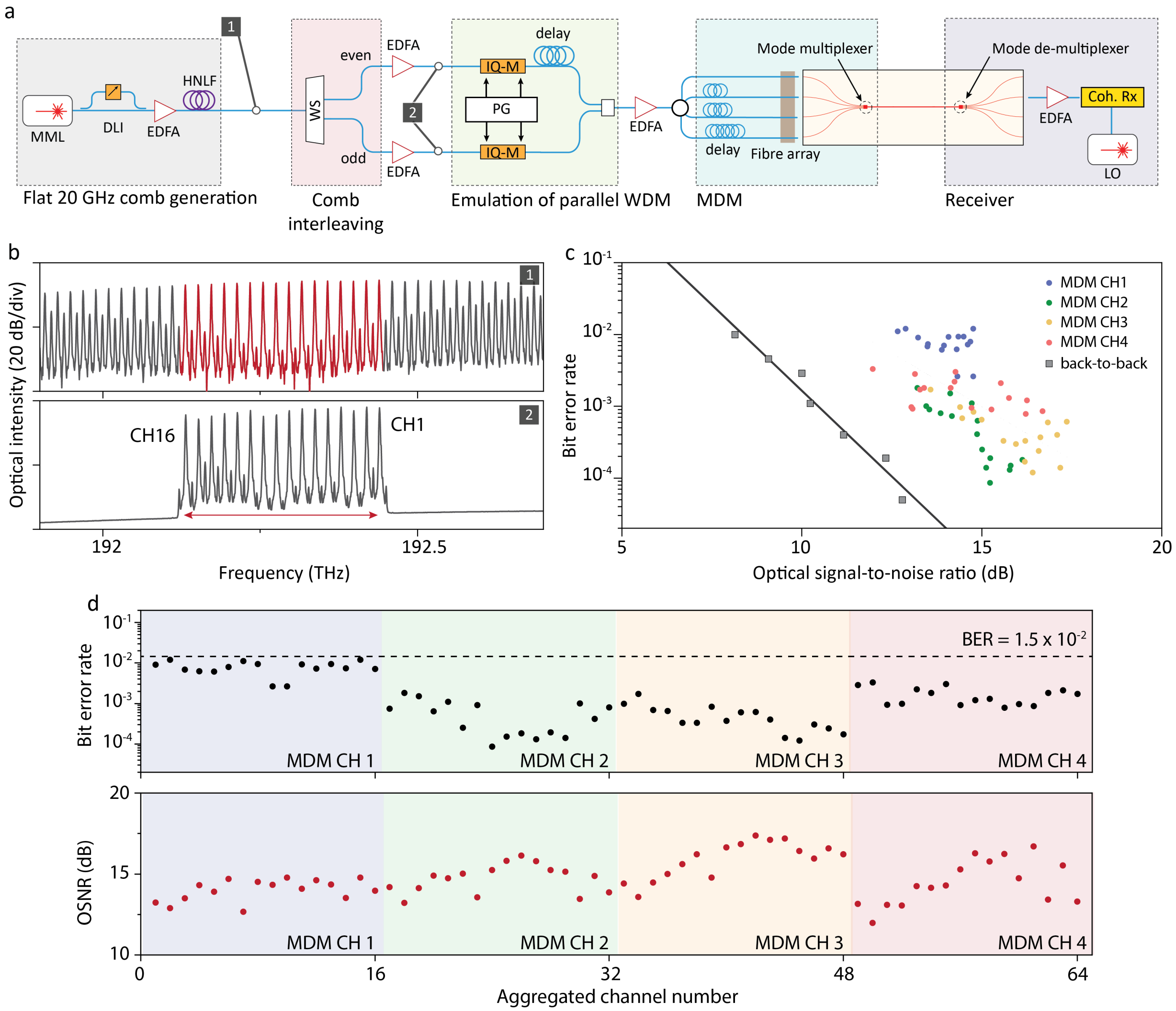

FIG. S7. **WDM - MDM data transmission using a 20-GHz spacing mode-locked laser.** **(a)** Schematic of experimental setup. **(b)** Measured optical spectra of flattened 20-GHz frequency comb (top) and selectively filtered comb tooth after interleaving and amplification (bottom). **(c)** Measured BER versus optical signal-to-noise ratio of all 64 channels as well as back-to-back test case. **(d)** Top: measured BERs of the transmitted data channels along with BER threshold when applying forward error correction with 20 % overheads (dashed black line), Bottom: Optical signal-to-noise ratio (OSNR) of the transmitted channels.

Mode-locked laser source In Fig.S7, a mode-locked laser (Lumentum, repetition rate: 10-GHz) generates a frequency comb. The frequency comb is passed through a delay line interferometer (free-spectral-range: 20-GHz) to increase the optical comb spacing. Then the 20-GHz comb is passed through a highly nonlinear fibre (HNLF) for spectral flattening and broadening[4]. The frequency lines of the optical comb are selectively filtered and de-interleaved into 'odd' and 'even' carriers by a programmable filter (Finisar WaveShaper). The 'odd' and 'even' carriers are separately amplified using C-band EDFAs, and routed to in-phase/quadrature modulators which are driven by arbitrary-waveform generators using quadrature phase-shift keying (at a symbol rate of 10-Gbd). The data channels are decorrelated by 50 symbols delays and combined into a standard single-mode fibre. Next we boost the power of all of the data channels using an EDFA and simultaneously launch them into the four input ports of the MDM chip using a fibre array (single-mode fibres with 127 μm spacing). Output coupling is performed with a tapered lensed fibre aligned to one MDM output waveguide at a time, and the received signal is characterized using intradyne coherent detection with electrical filtering and a tunable external cavity laser as a local oscillator. Fig.S7 shows the experimental setup and optical spectra of MLL sources.

The bit error rates (BERs) of the data transmission experiment are presented in Fig.S7c-d. The BERs of all the channels are under 1.5×10^{-2}, the limit of 20 % hard-decision forward error correction[5], enabling fault tolerant communication using error correcting codes. In this experiment, the larger BER of MDM channel 1 is likely due to mode cross-talk as well as channel-dependent optical loss. It is also important to note that the data rate we present is not limited by the MDM device; in this experiment we use 320 GHz (2.6 nm) of the device's 3.1 THz (25 nm) 3-dB bandwidth.

IV. FREE-SPACE DATA TRANSMISSION EXPERIMENT

Optical signals were generated using a CW tunable laser, passed through a circulator, and modulated with an amplitude electro-optic modulator. The modulator was driven by a data generated by micro-controller and electronic amplifier. To recover from modulator losses and to compensate for extraneous losses in the free space system, the signal was amplified with a fibre amplifier before coupling to the transmitter chip. The amplified beam passes through a polarization controller, free space coupler, and 50/50 beamsplitter and is focused onto input grating couplers through a 100× nIR objective lens (Mitutoyo). Light that is coupled out of the multimode output grating passes through the beamsplitter a second time and is collimated with a 200 mm focal length lens. This beam is sent approximately 0.5 m to another beamsplitter, 200 mm lens, and 100× objective that focus the multimode beam onto the multimode grating of the Rx chip. The output of the Rx chip is coupled out once more from the 100× objective and coupled to a single-mode fibre. 10 % of the light from the laser is picked off with a directional coupler, passed through a polarization controller, and 1 % of this light is coupled to the output fibre. The homodyne signal is read using a photodiode and a custom electric amplifier chain fed to an oscilloscope. Fig.S8-S9 show the experimental setup, free-space data transmission, and measured optical mode profiles at multiple wavelengths.

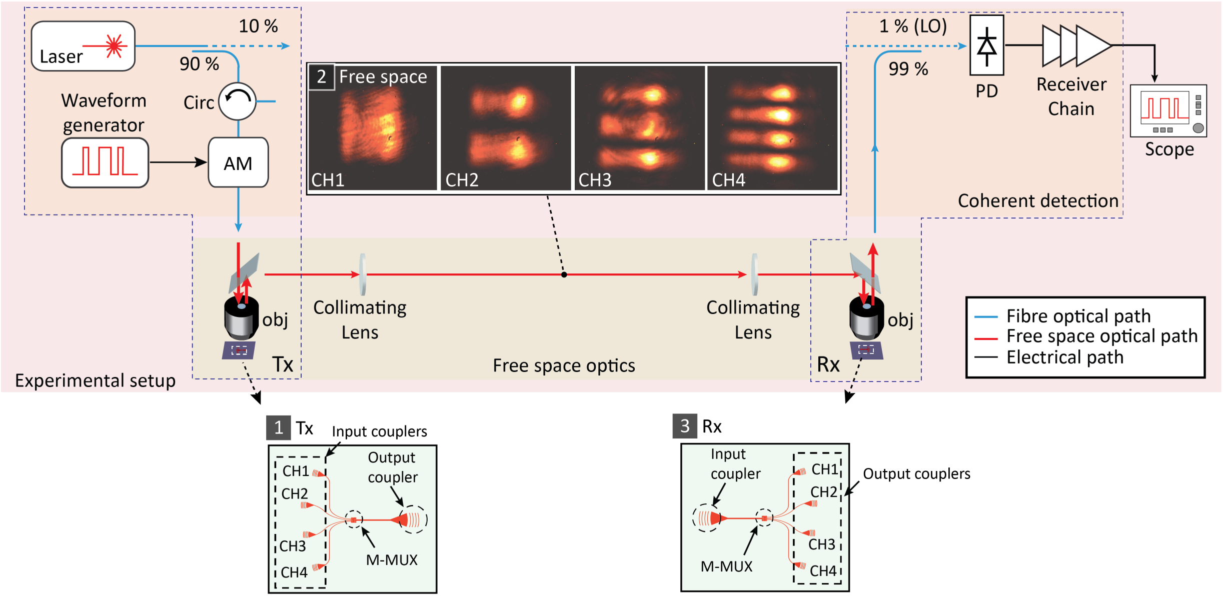

FIG. S8. **Free-space chip-to-chip optical link.** A CW laser is modulated with on-off-keying data using an amplitude modulator, and this data signal is coupled to the Tx input grating couplers. The Tx chip emits multi-channel MDM data to free-space using an inverse-designed coupler (Inset 1: schematic of the chip layout, Inset 2: mode profile in free-space between Tx and Rx chips). The free-space modes are projected onto an MDM demultiplexing device in the Rx chip that separates each mode into a single-mode output (Inset 3). The transmitted data are detected using a coherent receiver with the CW laser as the local oscillator (LO). Infrared images are measured at 1525 nm.

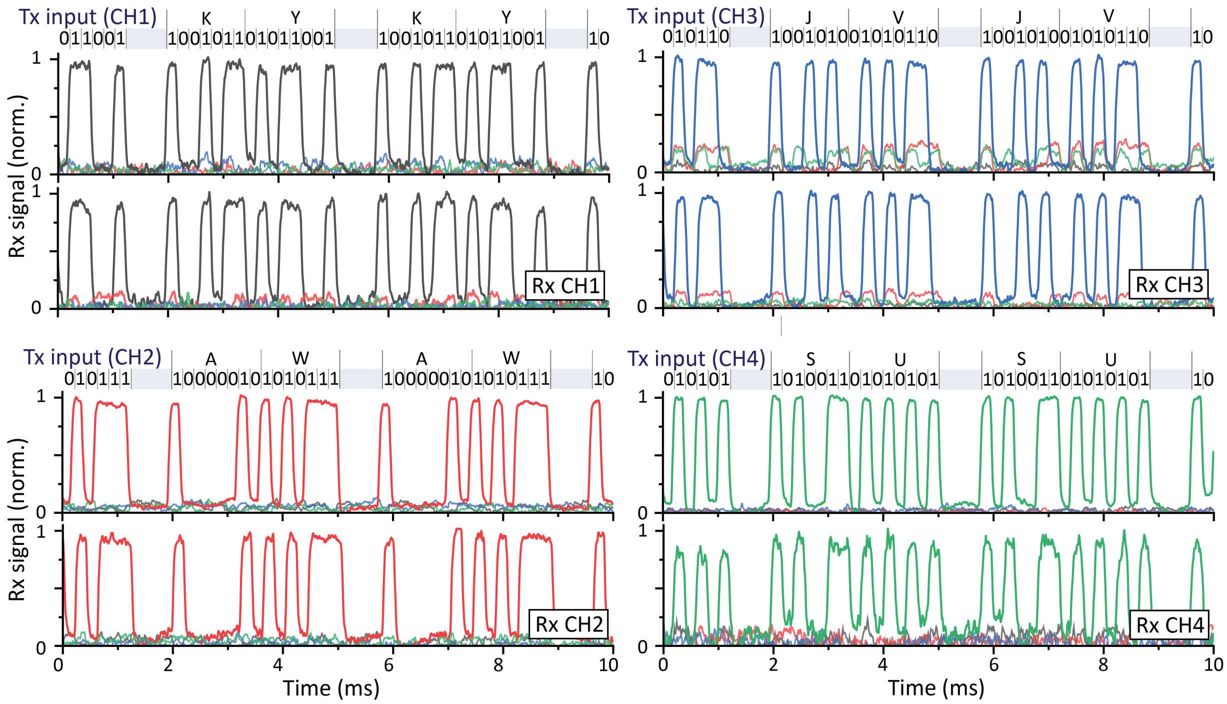

FIG. S9. **Chip-to-chip data transmission.** Received data traces at each Rx output grating coupler (Top: 1525 nm, Bottom: 1540 nm). The signal crosstalk at Rx CH3 trace is attributed to the effects of channel-dependent optical link insertion loss, the limited size aperture, and misalignment.

V. FOUR SPATIAL MODE RECTANGULAR CORE FIBRE

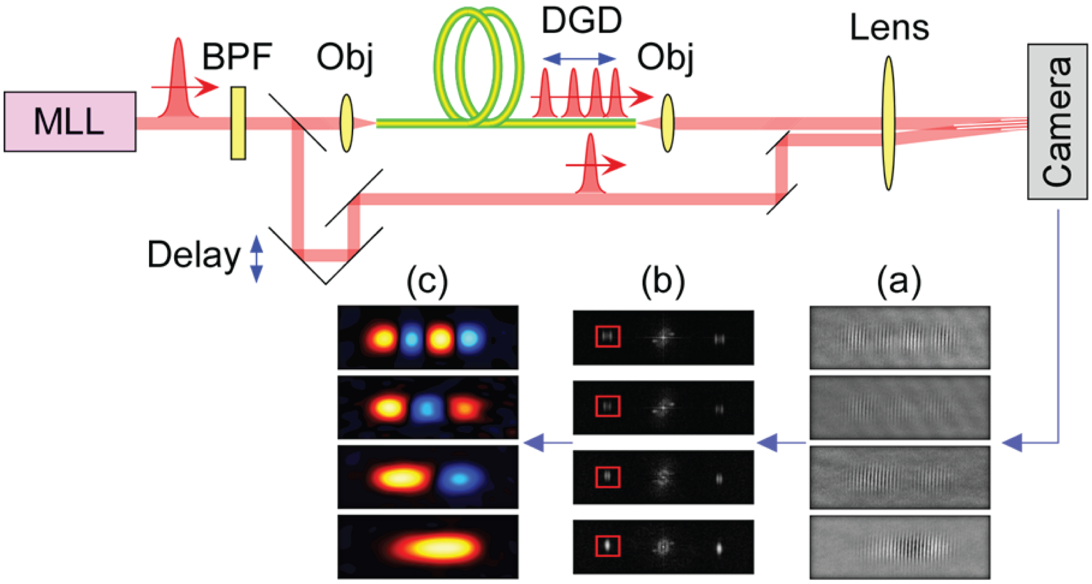

FIG. S10. **Four spatial mode rectangular core fiber.** Experimental setup for imaging fiber modes using low-coherence interferogram analysis. Short optical pulse from a mode-locked laser at center wavelength of 1.55 μm is focused with an objective (Obj) into the rectangular core fiber and excites all its spatial modes. (An optional band-pass filter (BPF) is used to increase the coherence lengths). At the fibre's distal end, the modes acquire differential group delays (DGD). A copy of the excitation pulse is timed for co-incidence onto an infrared camera, obtaining an interferogram of one mode at a time, inset (a). The mode profiles are extracted following a Fourier transform and selection of the off-axis diffracted signal (inset (b)), and inverse Fourier transform to reveal the mode profile, inset (c).

---

[1] Piggott, A. Y. *et al.* Inverse-designed photonics for semiconductor foundries. *ACS Photonics* **7**, 569–575 (2020).
[2] Idjadi, M. H. & Aflatouni, F. Nanophotonic phase noise filter in silicon. *Nature Photonics* **14**, 234–239 (2020).
[3] Zhang, Y. *et al.* A compact and low loss y-junction for submicron silicon waveguide. *Optics Express* **21**, 1310–1316 (2013).
[4] Ziyadi, M. *et al.* Tunable optical correlator using an optical frequency comb and a nonlinear multiplexer. *Optics Express* **22**, 84–89 (2014).
[5] Ryf, R. *et al.* 32-bit/s/Hz spectral efficiency wdm transmission over 177-km few-mode fiber. In *Optical fiber communication conference*, PDP5A–1 (Optical Society of America, 2013).
